# FePc/Mxene-Modified Electrode as a Highly Sensitive Sensing Platform for the Detection of Hg^2+^ in a Water Environment

**DOI:** 10.3390/nano16120708

**Published:** 2026-06-09

**Authors:** Cheng Yin, Zhang Luo, Chen Wen, Tingting Hu, Dandan Liu, Hao Peng, Huilai Liu, Xing Chen

**Affiliations:** 1 School of Resources and Environmental Engineering, Anhui Water Conservancy Technical College, Hefei 231603, China; yc@ahsdxy.edu.cn (C.Y.); ldd@ahsdxy.edu.cn (D.L.); 2 School of Resources and Environmental Engineering, Hefei University of Technology, Hefei 230009, China; 19397225192@163.com (C.W.); htting0212@163.com (T.H.); 3 School of Chemistry, Nanjing University, Nanjing 210023, China; 602025250036@smail.nju.edu.cn; 4 CCCC Yangtze River Construction Development Group Co., Ltd., Chongqing 400700, China; halepeng@163.com; 5 Chongqing Yufa Hydraulic Research Institute Co., Ltd., Chongqing 400010, China

**Keywords:** electrochemical sensor, Hg^2+^ detection, square-wave anodic stripping voltammetry

## Abstract

Inorganic mercury ions (Hg^2+^) are highly toxic, posing a threat to aquatic ecosystems and human health. In this study, iron phthalocyanine (FePc) was anchored onto the surface of MXene via a self-assembly strategy to construct an FePc/MXene-x (F/M-x) heterostructure. Characterization by scanning electron microscopy (SEM), transmission electron microscopy (TEM), X-ray photoelectron spectroscopy (XPS), and nitrogen adsorption–desorption (BET) confirmed that the high specific surface area and good conductivity of MXene effectively inhibited FePc aggregation and increased the exposure of active sites. The F/M-x composite was then modified onto a glassy carbon electrode (GCE) to fabricate an electrochemical sensor, and the detection performance for Hg^2+^ was evaluated using square-wave anodic stripping voltammetry (SWASV). Under optimized conditions (pH = 5.0, accumulation at −1.2 V for 180 s), the F/M-100/GCE exhibited a linear range of 0.1–1.0 μM, a sensitivity of 19.02 μA/μM, and a detection limit of 5.9 nM. The sensor showed good anti-interference ability against coexisting metal ions such as Cd^2+^, Cu^2+^, and Pb^2+^, with a batch-to-batch RSD of 2.03% and a long-term stability RSD of 2.49%. Spike recovery experiments in real water samples (lake water and groundwater) verified the accuracy of the method. This study provides a new electrochemical platform for the rapid detection of trace Hg^2+^ in water environments.

## 1. Introduction

With the development of human industry, the problem of water environment pollution has become increasingly prominent [1]. The discharge of industrial and agricultural wastewater and domestic sewage has made drinking water safety one of the serious challenges facing humanity today. Heavy metal ions in water are difficult to degrade naturally, continuously accumulating in water bodies, plants, and animals, and eventually entering the human body through the food chain, causing toxic effects and inducing various diseases [2]. If the mercury exposure level in human blood exceeds the recommended level (5 × 10^−7^ M), it may lead to permanent health damage [3]. Mercury exists in the environment mainly in the forms of elemental mercury (Hg^0^), inorganic mercury (Hg^2+^), and organic mercury (CH_3_Hg^+^, C_2_H_6_Hg^+^, C_6_H_6_Hg^+^) [4]. Among these, inorganic mercury ion is one of the most toxic heavy metal species in nature; excessive concentrations can cause serious health problems such as kidney and respiratory failure, gastrointestinal damage, neurological disorders, and impairments in language, hearing, and motor functions [5]. Therefore, it is of great significance to achieve accurate, rapid, and highly sensitive detection of Hg^2+^.

Currently, common analytical methods for detecting Hg^2+^ in the environment mainly include laboratory-based spectroscopic techniques such as atomic absorption spectrometry and inductively coupled plasma optical emission spectrometry/mass spectrometry [6,7]. These methods generally suffer from complicated operation, tedious sample pretreatment, high cost, and a tendency to cause secondary contamination during the detection process, making it difficult to meet the demand for rapid on-site monitoring of Hg^2+^ [8,9]. In contrast, electrochemical detection methods have attracted widespread attention due to their high sensitivity, simplicity of operation, low cost, and ease of integration [10,11]. The performance of electrochemical sensors mainly depends on the electrode modification material. Chemically modified electrodes, obtained by functionalizing the conductive substrate, exhibit significantly better performance than unmodified bare electrodes [12]. Nanomaterials, characterized by large surface area, strong adsorption capacity, and high surface activity, have been widely used in detection fields such as immobilization of biomolecules, signal amplification, and enhancement of target analyte enrichment processes [13]. Through functionalization and assembly, these materials can be easily modified onto electrode surfaces to improve the sensitivity and selectivity of pollutant molecule detection. For example, Lo et al. prepared biochar from sugarcane bagasse via slow pyrolysis at 500 °C and loaded it with silver nanoparticles to construct a novel composite electrode material (GC-SCBB@Ag). This electrode exhibited excellent electrochemical detection performance for Hg^2+^, with a detection limit as low as about 1 ppb and a good linear response [14]. Liu et al. synthesized a Cu-MOFs@MnO_2_ composite via a facile method and functionalized it on a screen-printed carbon electrode (SPCE), establishing an effective sensing interface. The results showed that the sensor achieved a detection limit of 30.07 nM for Hg^2+^ and maintained high selectivity and reproducibility in complex matrices [15].

As a novel type of two-dimensional transition metal carbide/nitride, MXene has attracted extensive attention from researchers in the fields of energy, catalysis, and environment due to its excellent electrical conductivity, photothermal conversion effect, and antibacterial properties [16]. However, pure MXene materials have relatively limited functionalities and poor electrocatalytic performance, which restrict their application in electrochemistry [17]. To address these issues, researchers have prepared MXene-based composites and utilized the synergistic effects among different components in heterostructures to obtain MXene-based composites with superior performance [18]. For example, Wang et al. developed an ultrasensitive electrochemical sensor based on an amino-functionalized MXene and bimetallic metal–organic framework nanocomposite (MXene-NH_2_@CeFe-MOF-NH_2_). The results showed that this composite possessed high conductivity, a large electroactive surface area, and a fast electron transfer rate, significantly enhancing the sensor‘s response current and detection efficiency [19]. Iron phthalocyanine (FePc) has attracted attention due to its unique FeN_4_ active sites and low reaction energy barrier, but it suffers from poor conductivity and easy aggregation, and the planar symmetric structure and charge distribution of FeN_4_ are unfavorable for the adsorption and catalysis of pollutant molecules [20]. To optimize the molecular structure of FePc, new strategies need to be explored, among which the introduction of a support catalyst is considered an effective approach [21]. Therefore, using MXene as a support to incorporate iron phthalocyanine molecules is expected to enhance the inherent catalytic activity of FePc.

In this study, FePc was loaded onto MXene to synthesize a series of FePc surface-loaded MXene composites. The microstructure of the synthesized materials was analyzed using characterization techniques such as scanning electron microscopy (SEM), high-resolution transmission electron microscopy (HRTEM), X-ray diffraction (XRD), and X-ray photoelectron spectroscopy (XPS). Furthermore, this series of composites was modified onto the surface of a glassy carbon electrode (GCE) to construct an electrochemical sensor. After optimizing the detection conditions, the sensor was evaluated via electrochemical methods for its sensitivity, limit of detection, anti-interference capability, repeatability, and stability in the analysis of Hg^2+^ in water environments, and its feasibility for detecting Hg^2+^ in real water samples was also verified.

## 2. Materials and Methods

### 2.1. Chemicals and Materials

Ti_3_AlC_2_ MAX phase was purchased from Foshan Xinxi Technology Co., Ltd., Foshan, China. Iron phthalocyanine (FePc), hydrofluoric acid (HF), dimethyl sulfoxide (DMSO), sodium borohydride (NaBH_4_), disodium hydrogen phosphate (Na_2_HPO_4_), potassium dihydrogen phosphate (KH_2_PO_4_), glacial acetic acid (C_2_H_4_O_2_), anhydrous sodium acetate (NaC_2_H_3_O_2_), N,N-dimethylformamide (DMF), citric acid (C_6_H_9_O_7_), and sodium citrate (C_6_H_5_Na_3_O_7_) were all purchased from Sinopharm Chemical Reagent Co., Ltd., Shanghai, China. The supporting electrolytes described in this study were prepared as follows: 0.1 M acetate buffer (ABS) was prepared by mixing 0.1 M C_2_H_4_O_2_ and NaC_2_H_3_O_2_ solutions; 0.1 M phosphate buffer (PBS) was prepared by mixing 0.1 M Na_2_HPO_4_ and KH_2_PO_4_ solutions; 0.1 M citrate buffer (CPBS) was prepared by mixing 0.1 M C_6_H_9_O_7_ and C_6_H_5_Na_3_O_7_ solutions. All chemicals were used as received without further purification. All solutions in this work were prepared with ultrapure water.

### 2.2. Synthesis of F/M-x

MXene was prepared using a hydrofluoric acid (HF) etching method [22]. First, 1 g of Ti_3_AlC_2_ precursor was slowly added to 20 mL of 40 wt% HF solution and continuously stirred at room temperature for 24 h. After the reaction, the solid product was collected by centrifugation and repeatedly washed with deionized water until the pH of the supernatant exceeded 6, followed by drying at 60 °C. To achieve delamination, the obtained powder was dispersed in dimethyl sulfoxide (DMSO) and stirred for intercalation over 24 h. Subsequently, the mixture was ultrasonicated under a nitrogen atmosphere for 24 h to assist in delamination. Finally, the product was centrifuged, washed, and vacuum-dried to obtain mono- or few-layer MXene nanosheets (Figure S1).

Briefly, 1 g of MXene material and 50 mg of iron phthalocyanine (FePc) were each dispersed in 100 mL of N,N-dimethylformamide (DMF) and ultrasonicated for 1 h, respectively. Then, the FePc-DMF solution was added to the MXene-DMF dispersion, followed by further ultrasonication for 0.5 h. The resulting mixed solution was continuously stirred for 20 h to achieve the loading of FePc onto MXene (Figure S2). After the reaction, the F/M-x composite was collected by centrifugation, washed three times each with DMF and absolute ethanol, and then vacuum-dried at 60 °C overnight. The obtained sample was denoted as F/M-50. Following the same preparation procedure, only the feeding mass of FePc was varied (100 mg, 150 mg) to obtain composites with different FePc loadings, which were named F/M-100 and F/M-150, respectively.

### 2.3. Characterization

The crystal structure of the materials was analyzed using a fixed-target X-ray diffractometer (XRD, X-Pert PRO MPD, Panalytical, Almelo, The Netherlands). The morphology was characterized using a thermal field-emission scanning electron microscope (SEM, Gemini 500, Carl Zeiss, Oberkochen, Germany) and a transmission electron microscope (TEM, JEM-2100F, JEOL, Tokyo, Japan). The bonding configuration and valence states were analyzed using an X-ray photoelectron spectroscope (XPS, ESCALAB 250Xi, Thermo Fisher Scientific, Waltham, MA, USA). The specific surface area and pore structure were measured using an automatic surface area and pore size analyzer (BET, Autosorb-IQ3, Quantachrome, Boynton Beach, FL, USA).

### 2.4. Electrochemical Measurements

All electrochemical measurements were performed in a 10 mL electrolytic cell using a three-electrode system, and data were processed with a CHI 760E computer-controlled potentiostat electrochemical workstation. A bare GCE or modified GCE was used as the working electrode, a Pt electrode as the counter electrode, and a Ag/AgCl electrode as the reference electrode. Cyclic voltammetry (CV) and electrochemical impedance spectroscopy (EIS) were employed to characterize the interfacial properties of the F/M-x-modified GCE in a solution containing the [Fe(CN_6_)]^3−/4−^ redox probe. The CV measurements were conducted in the potential range from −0.2 V to 0.6 V at a scan rate of 0.1 V/s. The EIS parameters were as follows: frequency range 10^6^–1 Hz, open-circuit potential 0.20 V, and amplitude 5 mV. Square-wave anodic stripping voltammetry (SWASV) was used for the electrochemical detection of Hg^2+^ in 0.1 M NaAc-HAc buffer (pH 5.0), with a deposition potential of −1.2 V and a deposition time of 180 s. Other parameters for the electrochemical measurements were as follows: step potential 5 mV, amplitude 5 mV, pulse amplitude 50 mV, pulse width 50 ms, sampling width 40 ms, and pulse period 0.1 s. Unless otherwise stated, all experiments were performed under these fixed parameters.

### 2.5. Preparation of Modified Electrodes

To further expand the application of F/M-x composites for Hg^2+^ detection, electrochemical sensors were constructed using four materials: MXene, F/M-50, F/M-100, and F/M-150. First, the bare glassy carbon electrode (GCE) was pretreated to remove surface impurities, ensuring accuracy and repeatability. The pretreatment procedure involved taking 10 mg of Al_2_O_3_ powders with different particle sizes (1 μm, 0.3 μm, and 0.05 μm), evenly spreading them on a chamois leather pad, and then polishing the GCE in a figure-eight pattern until the electrode surface became mirror-like. The electrode was subsequently ultrasonically cleaned for three minutes in a mixed solution of HNO_3_/ethanol/deionized water (1:1:1, *v*/*v*/*v*) and then dried for later use. After the above pretreatment, the nanomaterials (MXene, F/M-50, F/M-100, and F/M-150) were modified onto the bare GCE surface using the drop-casting method.

To prepare the modified electrodes, 1 mg of each powder (MXene, F/M-50, F/M-100, and F/M-150) was accurately weighed and separately added to 1 mL of deionized water, followed by ultrasonication for 20 min to obtain homogeneous and stable suspensions. Then, 6 μL of each suspension was pipetted onto the bare GCE surface and allowed to dry naturally at room temperature, yielding the corresponding material-modified electrodes. Thus, the glassy carbon electrodes modified with MXene, F/M-50, F/M-100, and F/M-150 were obtained for the electrochemical detection of mercury ions.

## 3. Results and Discussion

### 3.1. Structural and Morphological Characterizations

**Figure 1:**
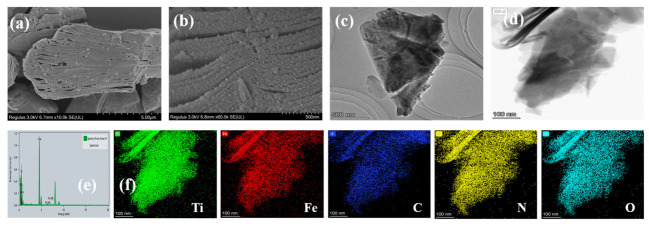
SEM images (**a**,**b**), TEM images (**c**,**d**), and EDS elemental mapping images (**e**,**f**) of F/M-100.

The morphology of the F/M-100 composite was characterized using SEM and TEM. The SEM images (Figure 1a,b) show that the prepared F/M-100 composite retained the accordion-like lamellar structure of MXene, with a slightly rough surface and reduced interlayer spacing. A small number of nanoparticles were observed to be attached to the MXene surface [23]. The TEM images further revealed the morphology of the F/M-100 composite. As can be seen from Figure 1c,d, F/M-100 exhibits a two-dimensional sheet-like structure, consistent with the SEM observations, and FePc molecules are tightly attached to the surface of MXene nanosheets, forming an uneven thin coating. Furthermore, elemental mapping results confirmed the presence and uniform distribution of Ti, C, O, Fe, and N elements in F/M-100 (Figure 1e,f).

**Figure 2:**
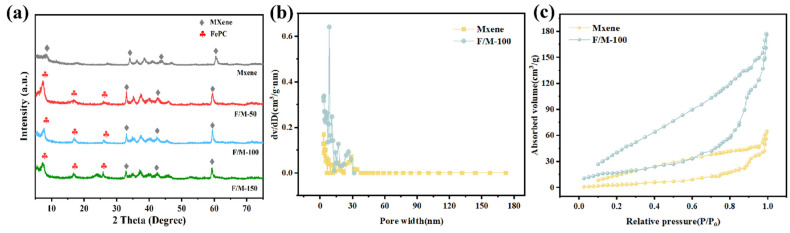
(**a**) X-ray diffraction patterns of MXene, F/M-50, F/M-100, and F/M-150 nanocomposites; N_2_ adsorption–desorption isotherms (**b**) and corresponding pore size distribution curves (**c**) of MXene and F/M-100 nanocomposites.

The phase composition of MXene and F/M-x nanocomposites was analyzed by XRD, and the results are shown in Figure 2a. The XRD pattern of pristine MXene exhibits three diffraction peaks at 2θ = 8.2°, 34.0°, 41.8°, and 61.6°, corresponding to the (002), (0010), (0012), and (110) crystal planes of MXene, respectively, indicating the successful synthesis of MXene nanosheets [24]. However, in the XRD patterns of the F/M-x composites, the intensities of the characteristic peaks of MXene changed with increasing FePc loading, suggesting that the introduction of FePc had a certain influence on the interlayer arrangement of MXene. Meanwhile, three new broad peaks appeared in the F/M patterns, centered at 7.8°, 17.6°, and 26.9°, corresponding to the (200), (310), and (422) crystal planes of FePc, respectively [25]. As the FePc loading increased from F/M-50 to F/M-150, the intensities of these three peaks gradually increased, indicating that FePc was successfully loaded onto the MXene nanosheets while maintaining its crystalline structure.

The effect of FePc incorporation on the specific surface area and pore structure of MXene was investigated using N_2_ adsorption–desorption isotherms and pore size distribution curves. From the pore size distribution curves (Figure 2b), it can be seen that pristine MXene exhibits a relatively narrow pore size distribution and low pore volume. In contrast, F/M-100 shows a stronger pore distribution peak in the range of 2–30 nm, with a significantly increased pore volume. This indicates that the loading of FePc effectively inhibits the stacking of MXene nanosheets and increases the number of mesopores and the pore volume of the material, thereby providing more active sites and mass transfer channels for adsorption–catalysis reactions, which is beneficial for enhancing catalytic performance [26]. Furthermore, the N_2_ adsorption–desorption isotherms of MXene and F/M-100 (Figure 2c) both exhibit typical Type IV isotherms with an obvious hysteresis loop in the medium-to-high relative pressure region, indicating that both materials are dominated by mesoporous structures. Compared with pristine MXene, the N_2_ adsorption capacity of F/M-100 is significantly increased, and its specific surface area is clearly larger than that of pristine MXene, demonstrating that the introduction of FePc effectively increases the specific surface area of the material.

**Figure 3:**
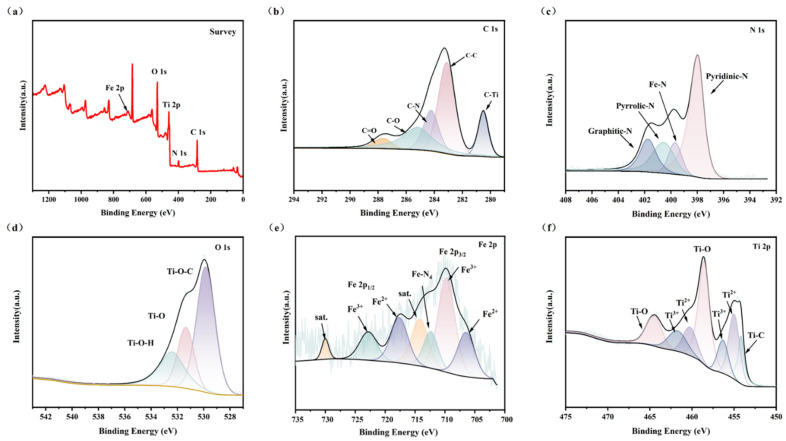
XPS spectra of F/M-100 nanomaterial: (**a**) survey spectrum, (**b**) C 1s, (**c**) N 1s, (**d**) O 1s, (**e**) Fe 2p, and (**f**) Ti 2p.

The elemental composition and surface chemical state of the F/M-100 nanocomposite were characterized by XPS, and the results are shown in Figure 3a. Five characteristic peaks were observed at binding energies of 735.9 eV, 454.8 eV, 530.6 eV, 401 eV, and 285.5 eV, corresponding to Fe 2p, Ti 2p, O 1s, N 1s, and C 1s, respectively, further confirming the successful introduction of heteroatoms (Fe, N) into MXene [27]. Figure 3b shows the high-resolution XPS spectrum of C 1s, which can be deconvoluted into five characteristic peaks at binding energies of 282.1 eV, 283.6 eV, 284.8 eV, 286.1 eV, and 288.4 eV, corresponding to C–Ti, C–N, C–C, C–O, and C=O bonds, respectively [28]. Figure 3c presents the high-resolution N 1s spectrum, which can be fitted into four peaks at binding energies of 398.6 eV, 399.7 eV, 400.7 eV, and 402.0 eV, assigned to pyridinic-N, Fe–Nx, pyrrolic-N, and graphitic-N species, respectively [29]. The Fe–Nx coordination structure may provide key active sites for adsorption–catalysis reactions. Figure 3d shows the high-resolution O 1s spectrum of F/M-100, with peaks at 530.0 eV, 531.3 eV, and 532.4 eV, corresponding to Ti–O–C, Ti–O, and Ti–OH bonds, reflecting the oxygen-containing functional groups and oxidation state of the MXene surface [30]. Figure 3e is the Fe 2p XPS spectrum. In the Fe 2p_3/2_ and Fe 2p_1/2_ spectra, the typical characteristic peaks at binding energies of 706.8 eV and 717.6 eV correspond to Fe^2+^, while those at 709.9 eV and 722.7 eV correspond to Fe^3+^. Moreover, a Fe–N_4_ coordination signal is observed at approximately 712.4 eV [20,31]. Figure 3f shows the high-resolution Ti 2p XPS spectrum, which can be fitted into multiple peaks corresponding to Ti–C, Ti^3+^, and Ti–O species [32]. The results indicate that MXene not only retains its layered structure during the composite formation but also undergoes partial surface oxidation and coordination interactions.

### 3.2. Electrochemical Performance of F/M-x-Modified Electrodes

**Figure 4:**
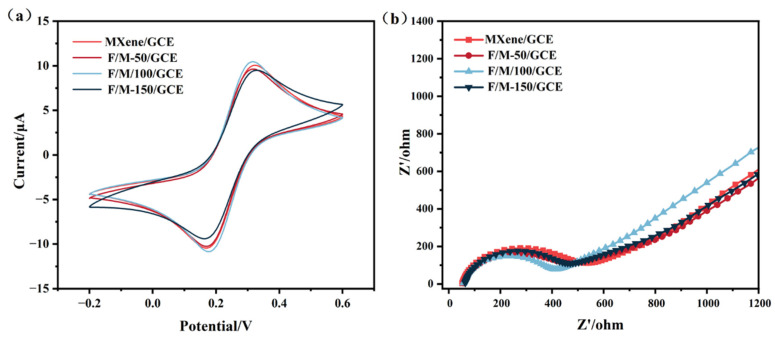
(**a**) Cyclic voltammetry curves and (**b**) electrochemical impedance spectra of MXene-, F/M-50-, F/M-100-, and F/M-150-modified electrodes in 5 mM [Fe(CN)_6_]^3−^/^4−^ (0.1 M) solution.

To investigate the electrochemical performance of the electrochemical interfaces constructed with MXene, F/M-50, F/M-100, and F/M-150 nanomaterials, they were characterized by CV and EIS in a solution containing a 5 mM [Fe(CN)_6_]^3−/4−^ redox probe. As shown in Figure 4a, all materials exhibited a symmetric redox peak at 0.213 V. Among them, MXene/GCE showed the lowest redox current, while the redox currents on the modified electrodes all increased, indicating that the composites were successfully attached. The highest peak current was obtained with the F/M-100 material, which is attributed to the synergistic effect between FePc and MXene. This synergy not only significantly increased the electroactive surface area of the modified electrode and promoted electron transfer within the material but also effectively enhanced the electron transfer efficiency at the electrode interface and the adsorption-enrichment ability for Hg^2+^, thereby substantially optimizing the electrochemical response for Hg^2+^ detection [33]. EIS can provide information on the electron transfer resistance of nanomaterial-modified electrodes. Figure 4b shows typical Nyquist plots of the four electrodes. The diameter of the semicircular portion represents the electron transfer resistance (R_et_) at higher frequencies, and the radius of the semicircle is inversely proportional to the charge transfer efficiency. The linear portion corresponds to the diffusion process at lower frequencies [34]. The F/M-100-modified GCE exhibited the lowest electron transfer resistance, which is due to the synergistic effect between FePc and MXene, effectively accelerating the electron transfer at the electrode/electrolyte interface and thus improving the electrical conductivity of the material, consistent with the CV data.

The active specific surface areas of MXene and F/M-100 were determined by varying the scan rate in an electrolyte solution containing 0.1 M KCl and 5 mM [Fe(CN)_6_]^3−/4−^. As shown in Figure S3, the peak current intensity increased with increasing scan rate. Moreover, the peak current intensity (I_p_) of F/M-100 exhibited a linear relationship with the square root of the scan rate, following the Randles–Sevcik equation (Equation (1)) [35]:

(1) $$I_{p}=2.69\times 10^{5}n^{3/2}AD^{1/2}v^{1/2}C_{0}$$

where *n* is the number of electrons transferred, *A* is the electroactive surface area, *D* is the diffusion coefficient, *v* is the scan rate, and *C*_0_ is the concentration of the [Fe(CN)_6_]^3−/4−^ probe. Here, *n* = 1, *D* = 7.6 × 10^−6^ cm^2^/s, and *C*_0_ = 5 × 10^−6^ M. The electroactive surface areas of the different modified electrodes were calculated accordingly. The electroactive surface areas of MXene/GCE and F/M-100/GCE were found to be 0.0069 cm^2^ and 0.0411 cm^2^, respectively (Figure S3). Comparing these values, the F/M-100 sensing interface exhibits a significantly larger electroactive surface area. The high metallic conductivity of MXene nanosheets constructs a fast electron transfer network. The uniform loading of FePc molecules onto the MXene scaffold effectively inhibits the self-stacking of the nanosheets, thereby exposing more active sites. This highly open and conductive heterostructure provides an extremely favorable physicochemical interface for the adsorption and electrochemical enrichment of Hg^2+^.

### 3.3. Optimization of Electrochemical Conditions

As shown in Figure S4, the electrochemical response signals for Hg^2+^ detection were measured in 0.1 M NaAc-HAc buffer solution (pH = 5.0) using GCEs modified with MXene, F/M-50, F/M-100, and F/M-150. The peak currents for Hg^2+^ detection followed the order F/M-100 > F/M-150 > F/M-50 > MXene. Compared with MXene and F/M-50, the F/M-100-modified electrode exhibited a significantly higher peak current for Hg^2+^, indicating that an appropriate amount of FePc effectively enhances the enrichment ability and electrocatalytic activity of the electrode toward Hg^2+^ while also significantly improving the interfacial electron transfer efficiency. Moreover, the peak current of the F/M-150-modified electrode showed a marked decrease, likely because an excess of FePc leads to aggregation of active sites and blockage of mass transfer channels in MXene, thereby weakening the sensing interface performance. Overall, the response current of F/M composites with different FePc loadings for Hg^2+^ detection exhibited a trend of first increasing and then decreasing, with F/M-100 showing the optimal electrochemical response, representing the best balance of synergy between MXene and FePc. Table S1 presents a comparison of the analytical performance of the as-prepared FePc/MXene (F/M-100) sensor with other representative electrochemical sensors for Hg^2+^ detection reported in the literature.

To further investigate the optimal conditions for Hg^2+^ detection, the electrochemical parameters were optimized using the F/M-100-modified GCE, including buffer type, pH, accumulation time, and accumulation potential (Figure S5). All optimization experiments were performed using a 0.5 μM Hg^2+^ solution. Figure S5a shows the Hg^2+^ response currents in three different buffers (0.1 M ABS, 0.1 M PBS, and 0.1 M CBPS) at pH 5.0. It can be observed that Hg^2+^ exhibited the highest detection sensitivity in ABS at pH 5.0; therefore, subsequent experiments were conducted in 0.1 M NaAc-HAc buffer. The pH of the ABS buffer was then adjusted within the range of 3.0–6.0 to study its effect on the Hg^2+^ signal. The best detection performance was obtained at pH 5.0. Under strongly acidic conditions, H^+^ is preferentially reduced to hydrogen, occupying active sites on the electrode and competing with Hg^2+^ reduction, thereby decreasing the peak response (Figure S5b).

The response current of F/M toward Hg^2+^ was also influenced by both accumulation potential and accumulation time, both of which are critical factors. The optimization of accumulation time is shown in Figure S5c. The time was gradually increased from 90 s to 210 s to evaluate its effect on the evolution of the Hg^2+^ redox signal. The peak current for Hg^2+^ detection first increased and then decreased with increasing accumulation time, reaching a significant peak at 180 s, after which the response current gradually declined with a further increase in accumulation time. The electrochemical response signal of F/M for 0.5 μM Hg^2+^ in the potential range from –2.1 V to –0.3 V is displayed in Figure S5d. At accumulation potentials of –2.1 V and –1.8 V, the current values were relatively low. This is attributed to the fact that an excessively low potential causes H^+^ to be reduced to H_2_, and the resultant bubbles on the electrode surface block active sites, thus affecting the mass transfer of Hg^2+^ ions and eventually reducing the efficiency of mercury deposition, leading to a weaker anodic stripping signal. As the accumulation potential increased, the response current gradually rose, reaching a maximum at −1.2 V, indicating the best detection performance. Based on the above experiments, the optimal conditions for Hg^2+^ detection are summarized as follows: 0.1 M NaAc-HAc buffer, pH 5.0, accumulation time 180 s, and accumulation potential −1.2 V.

### 3.4. Electrochemical Determination of Hg^2+^

**Figure 5:**
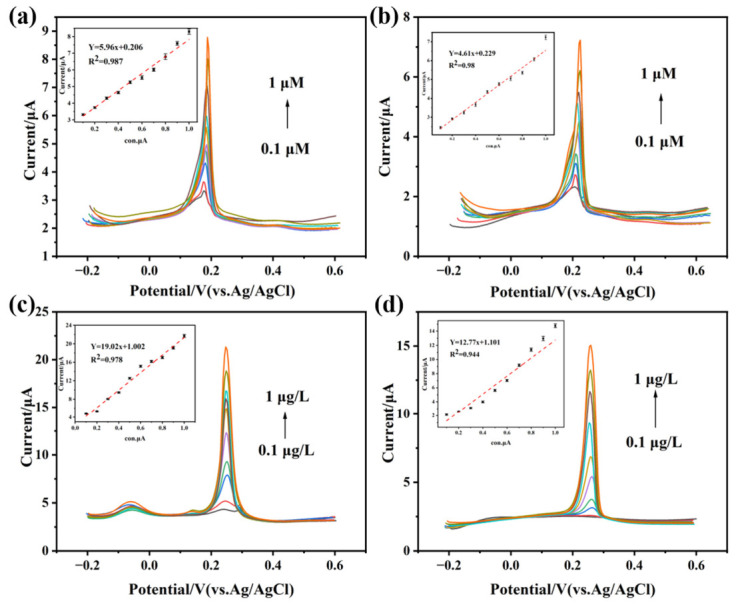
SWAS responses and calibration curves of response currents for Hg^2+^ over the concentration range of 0.1–1.0 μM at GCEs modified with MXene (**a**), F/M-50 (**b**), F/M-100 (**c**), and F/M-150 (**d**).

Figure 5 shows the anodic stripping voltammetry curves for detecting different concentrations of Hg^2+^ (0.1–1.0 µM) under optimized experimental parameters using square-wave anodic stripping voltammetry (SWASV) with MXene/GCE, F/M-50/GCE, F/M-100/GCE, and F/M-150/GCE as working electrodes, respectively. As shown in Figure 5a, the bare MXene-modified electrode exhibited a certain response to Hg^2+^, with the stripping peak current gradually increasing as the Hg^2+^ concentration increased. After introducing iron phthalocyanine (FePc), the peak current of F/M-50/GCE (Figure 5b) significantly increased, reflecting the specific coordination ability of FePc toward Hg^2+^. Upon further increasing the FePc ratio to F/M-100 (Figure 5c), an excellent linear relationship was observed between the electrochemical response current and Hg^2+^ concentration: I/μA = 1.002 + 19.02X C/μA/μM, R^2^ = 0.978. The sensitivity (S) of the modified electrode for Hg^2+^ detection is given by the slope of the linear fit equation. The limit of detection (LOD) and limit of quantification (LOQ) were calculated using the following formulas (Equations (2) and (3)) [36]:

(2) LOD = 3σ/S

(3) LOQ = 10σ/S

where σ is the signal-to-noise ratio. The LOD and LOQ of this modified electrode for Hg^2+^ detection were 5.9 nM and 19.6 nM, respectively. In contrast, the response of the F/M-150/GCE (Figure 5d) decreased slightly, which may be attributed to excessive loading of iron phthalocyanine leading to material aggregation and a reduced effective specific surface area. The above results indicate that the F/M-100-modified electrode fully exploits the synergistic effect of the efficient coordination ability of iron phthalocyanine and the large specific surface area and excellent conductivity of MXene, thereby achieving highly sensitive detection of Hg^2+^.

### 3.5. Electrochemical Kinetic Analysis

In this study, the number of electrons (n) transferred during the electrochemical reaction of Hg^2+^ was estimated by electrochemical kinetic methods. The experiments were carried out in a 0.1 M NaAc-HAc buffer solution (pH = 5.0) containing 10 μM Hg^2+^, with scan rates ranging from 0.05 to 0.4 V·s^−1^. Figure S6 shows the variation in the current with scan rate for different modified electrodes (MXene/GCE and F/M-100/GCE). The results indicate that the redox peak currents increase with increasing scan rate. The peak currents (I_pa_ and I_pc_) shown in the inset exhibit a good linear relationship with the square root of the scan rate, suggesting that the redox process is controlled by diffusion on the electrode surface. Meanwhile, the oxidation peak potential (E_p_) on the F/M-100/GCE shifts positively with increasing scan rate, and a good linear correlation is observed between E_p_ and the natural logarithm of the scan rate (ln(ν)). Based on the Laviron equation, the kinetic parameters of the electrochemical reaction and the number of transferred electrons can be further analyzed (Equation (4)):

(4) $$E_{p}=\frac{RT}{\alpha nF}ln\frac{k^{0}RT}{\alpha nF}-\frac{RT}{\alpha nF}ln\nu$$

where *R* is the gas constant, *T* is the absolute temperature, α is the charge transfer coefficient, *k*^0^ is the standard rate constant, *n* is the number of electrons transferred in the electrochemical reaction, and *F* is the Faraday constant. According to the above relationship, the number of transferred electrons for the MXene and F/M-100-modified electrodes was calculated to be 1.05 and 1.63, respectively. The electron transfer numbers we measured (1.05 and 1.63) are lower than the expected value for the theoretical two-electron process of Hg^2+^/Hg^0^. The main reasons may be as follows: (1) Under certain conditions (e.g., at the initial stage of the reduction wave or during the first reduction scan), a small amount of Hg(I) intermediate can be detected, which is not completely further reduced, resulting in an experimentally measured n value lower than the theoretical value. (2) Due to the formation of mercury droplets or a mercury film, the diffusion behavior deviates from the ideal model, and the measured n values typically range from 1.5 to 1.8 [37,38]. This kinetic parameter provides a quantitative basis for understanding the electrocatalytic mechanism of Hg^2+^ at the F/M-100-modified electrode.

**Figure 6:**
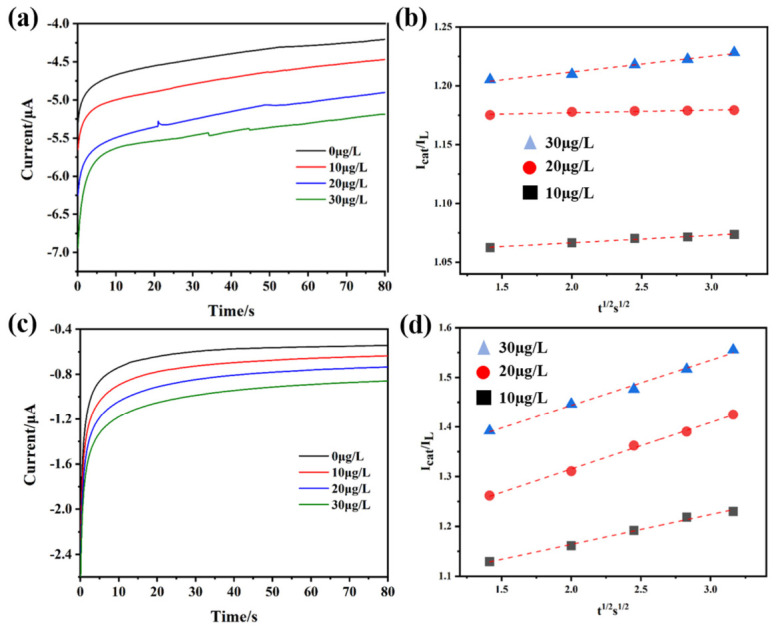
Chronoamperometric responses of (**a**) MXene- and (**c**) F/M-100-modified GCEs upon addition of different concentrations of Hg^2+^ (0–30 µg/L) and (**b**,**d**) the corresponding plots of I_cat_/I_L_ and t^1/2^.

To explore the differences in electrocatalytic ability of the four sensing interfaces toward Hg^2+^, their electrocatalytic parameters were evaluated by chronoamperometry (CA). Figure 6 shows the current–time relationships for different concentrations of Hg^2+^ at MXene- and F/M-100-modified GCEs. The electrolyte solution for the CA system was 0.1 M HAc-NaAc, and Hg^2+^ was added at concentrations of 0, 10, 20, and 30 μg/L. It can be seen that the reaction current changed upon the addition of Hg^2+^, indicating that the modified electrochemically sensitive interfaces possess a certain electrocatalytic effect toward Hg^2+^. Moreover, for both MXene and F/M-100-modified GCEs, the ratio I_L_/I_cat_ exhibited a linear relationship with the square root of time. Therefore, the catalytic rate constant (K_cat_) for the different reaction interfaces was estimated using the Cottrell equation (Equation (5)) [39]:

(5) $$\frac{I_{cat}}{I_{L}}={\left(\pi K_{cat}C_{0}t\right)}^{1/2}$$

where *I_cat_* is the response current of the reaction system with the addition of Hg^2+^, *I_L_* is the response current of the reaction system without the addition of Hg^2+^, *C*_0_ is the concentration of Hg^2+^, and *t* is the reaction time. The calculated electrocatalytic rate constants for the MXene- and F/M-100-modified GCEs were 1.07 × 10^−3^ s^−1^/µM and 2.22 × 10^−3^ s^−1^/µM, respectively. The results indicate that the F/M-100 GCE exhibits better electrocatalytic performance toward Hg^2+^ than the MXene material, which is consistent with previous findings.

### 3.6. Anti-Interference Performance

Considering the complexity of natural water matrices, this study further investigated the signal interference from common coexisting metal ions on the constructed electrochemical sensing interface for Hg^2+^ detection. Based on groundwater quality standards and environmental survey data, experiments were conducted using the maximum concentration levels of representative metal ions typically found in groundwater. In a 0.1 M acetate buffer solution (pH 5.0) containing 0.5 μM Hg^2+^, Cd^2+^, Cu^2+^, Fe^3+^, Pb^2+^, Zn^2+^, and Bi^2+^, they were each added at a concentration of 1 μM. The electrochemical response of the F/M-100 GCE to Hg^2+^ was measured using square-wave anodic stripping voltammetry (SWASV) under an accumulation potential of −1.2 V and an accumulation time of 180 s.

**Figure 7:**
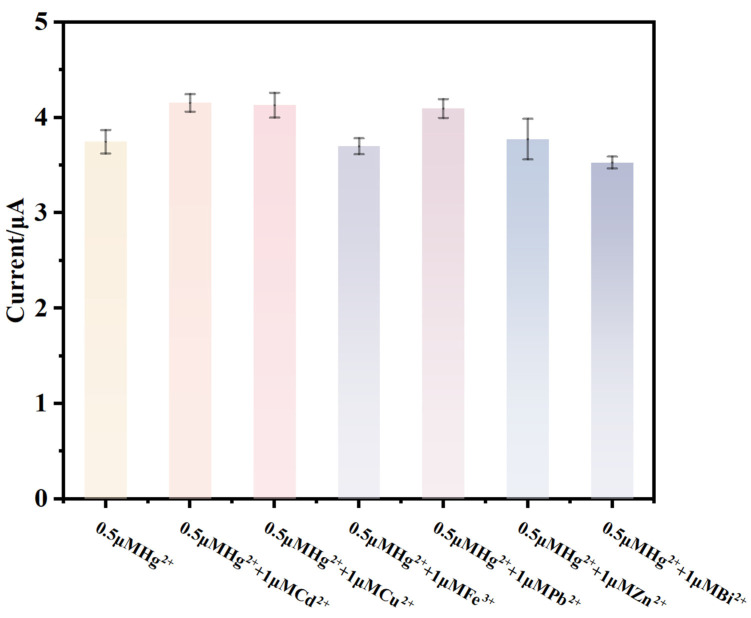
SWAV responses of 0.5 μM Hg^2+^ on F/M-100-modified GCE in the presence of different interfering substances. Considering the complexity of natural water components, the influence of common environmental metal ions on the Hg^2+^ signal at the constructed electrochemical sensing interface was investigated.

The results are shown in Figure 7. The addition of Fe^3+^, Zn^2+^, and Bi^2+^ caused a slight decrease in the stripping peak current of Hg^2+^, presumably due to ion competition effects on the electrochemically sensitive interface. Fe^3+^ consumes the applied potential and competes with Hg^2+^ for active sites on the electrode surface, while Bi^2+^ can form bismuth–mercury intermetallic compounds with deposited Hg^0^, altering the stripping potential and peak shape of Hg^0^ oxidation. The addition of Pb^2+^, Cu^2+^, and Cd^2+^ resulted in a slight enhancement of the Hg^2+^ response signal; the reason may be that Cu^2+^ can form Cu-Hg intermetallic compounds with Hg^2+^ on the electrode surface or within the mercury film, thereby altering the stripping potential and peak current of Hg^0^. Although Pb^2+^ and Cd^2+^ do not directly form compounds with Hg^2+^, they compete for active sites on the electrode surface, affecting the enrichment efficiency of Hg^2+^.

Quantitative analysis indicates that the influence rates of Fe^3+^, Zn^2+^, and Bi^2+^ on the Hg^2+^ detection current are all below 5%, with the interference from Fe^3+^ and Zn^2+^ being almost negligible. The influence rates of Cd^2+^, Cu^2+^, and Pb^2+^ are all around 10%, which are within acceptable interference levels, demonstrating that the sensing interface constructed with F/M-x possesses good anti-interference performance for Hg^2+^ detection.

There are various potential interferents in real water environments, including Na^+^, K^+^, Ca^2+^, Cl^−^, NO_3_^−^, SO_4_^2−^. Therefore, a comprehensive evaluation of their influence on the sensor is of great importance (Figure S7). The effects of 1 µM of Na^+^, K^+^, and Ca^2+^ and 1 µM of Cl^−^, NO_3_^−^, and SO_4_^2−^ on the detection signal of 0.5 μM Hg^2+^ were individually tested. The results showed that the signal variations were all within ±8%, indicating acceptable interference.

### 3.7. Repeatability and Reproducibility

**Figure 8:**
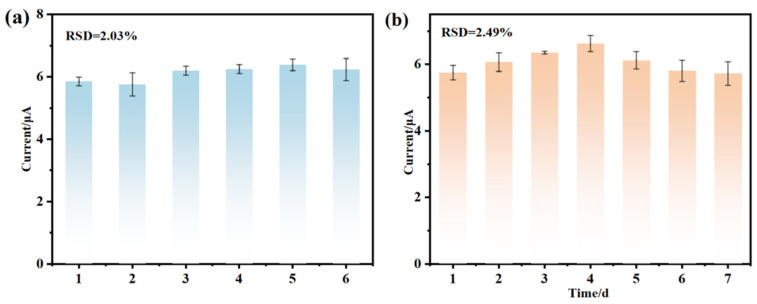
(**a**) SWAV responses of six F/M-100-modified electrodes to 0.5 μM Hg^2+^; (**b**) repeated detection of 0.5 μM Hg^2+^ over seven consecutive days using the F/M-100@PM-modified electrode.

The stability evaluation of a sensor is a key prerequisite for its practical application. Therefore, this study further examined the stability of the constructed sensing interface. The detection stability of the F/M-100-modified GCE for Hg^2+^ was systematically evaluated through repeatability and reproducibility experiments. First, six independent F/M-100-modified electrodes (E1–E6) were prepared, and their electrochemical responses were tested in a buffer solution containing 0.5 μM Hg^2+^. The results showed that the response currents of the electrodes were stable, with a low batch-to-batch relative standard deviation (RSD) of 2.03%, indicating minimal inter-electrode variation (Figure 8a).

The same F/M-100-modified GCE was continuously measured over a period of one week, and its stripping peak current for 0.5 μM Hg^2+^ was recorded at fixed time points. As shown in Figure 8b, the peak currents obtained from seven measurements exhibited very little fluctuation, with an RSD of only 2.49%, demonstrating that the electrode possesses excellent long-term stability and reproducibility for Hg^2+^ detection. These outstanding repeatability and reproducibility results indicate that the F/M-100-modified GCE has a reliable Hg^2+^ detection capability in practical analysis. The same electrode was left undisturbed at room temperature. The operational results after two weeks of repeated SWASV scans remained stable, with an RSD of 3.97% (Figure S8).

### 3.8. Analysis of Natural Water Samples

To evaluate the reliability of the F/M/GCE sensor for real water sample analysis, water samples were collected from HuBing Pond and groundwater from a certain area in Anhui Province. First, a certain amount of water from HuBing Pond was filtered through a 0.45 μm membrane to remove large insoluble particles that might affect the experimental results. The filtered water sample was mixed with 0.1 M HAc-NaAc buffer at a volume ratio of 1:9, adjusting the solution pH to 5.0. Under the optimized experimental conditions, the F/M-100-modified GCE was used as the working electrode to measure the SWASV response of the sensor to Hg^2+^ in both pure buffer and the buffer containing the groundwater sample. As shown in Figure S9, no obvious Hg^2+^ stripping peak appeared in the pure buffer solution, while the system containing groundwater exhibited a clear, symmetric stripping peak at approximately 0.2 V, and the curves from repeated tests showed good repeatability. The results indicate that the introduction of the real groundwater matrix did not significantly alter the stripping peak potential of Hg^2+^, and the peak current did not show obvious attenuation. Moreover, the sensor exhibited good anti-interference ability against the groundwater matrix, and no matrix effect affected the detection signal of the target analyte.

On this basis, the filtered and diluted groundwater samples were further analyzed by the standard addition method, and the results are shown in Figure S10. As the spiked concentration of Hg^2+^ increased from 0.2 μM to 1.0 μM (with a concentration gradient of 0.2 μM), the stripping peak current increased regularly with the concentration, and the peak shape remained stable. The inset shows the linear calibration curve of the peak current versus the spiked concentration. The linear regression equation is Y = 10.434X − 0.7053, with a correlation coefficient R^2^ = 0.9960, indicating that the sensor exhibits good linear response and quantitative capability for Hg^2+^ in the actual groundwater matrix. These results demonstrate that the SWASV response of the F/M/GCE sensor to Hg^2+^ is only minimally affected by the real water sample matrix, enabling stable detection of trace Hg^2+^ in natural water matrices and thus showing great application potential for Hg^2+^ analysis in environmental water samples.

## 4. Conclusions

In this study, a heterostructured nanocomposite of iron phthalocyanine (FePc) and two-dimensional transition metal carbide (MXene), denoted as F/M-x, was successfully constructed via a self-assembly strategy and employed as an electrode modification material to build an electrochemical sensing platform for the detection of ultra-trace Hg^2+^ in water environments. Systematic characterization confirmed that FePc molecules were uniformly anchored on the surface of MXene nanosheets, and the high specific surface area of MXene effectively inhibited the aggregation of FePc, significantly increasing the exposure of active sites. Electrochemical analysis revealed that the F/M-100-modified electrode exhibited excellent signal amplification in square-wave anodic stripping voltammetry (SWASV). Under optimized conditions (pH 5.0 acetate buffer, accumulation potential −1.2 V, accumulation time 180 s), the sensor showed a linear response range of 0.1–1.0 μM for Hg^2+^, a sensitivity of 19.02 μA/μM, and a low detection limit of 5.9 nM. The sensor also demonstrated good anti-interference ability against common coexisting metal ions (Cd^2+^, Cu^2+^, Pb^2+^, etc.), with a batch-to-batch repeatability RSD of 2.03% and a long-term stability RSD of 2.49%. Spike recovery experiments in real water samples verified its accuracy and reliability. Leveraging the synergistic effect between the M-N_4_ active centers of FePc and the highly conductive framework of MXene, this work provides an efficient sensing platform for the rapid and sensitive detection of trace Hg^2+^ in water environments and also offers a new design strategy for macrocyclic complex/2D nanocomposite interfaces.

## Data Availability

The data presented in this study are available on request from the corresponding author.

## Supplementary Materials

The following supporting information can be downloaded at: https://www.mdpi.com/article/10.3390/nano16120708/s1, Figure S1. Schematic illustration of the preparation of MXene nanocomposite. Figure S2. Schematic illustration of the preparation of FePc/MXene-x nanocomposite. Figure S3. Cyclic voltammetry curves of (a) MXene and (b) F/M-100 in 0.1 M KCl solution containing 5 mM [Fe(CN)_6_]^3−/4−^ at different scan rates (0.1–0.8 V/s), and (c) the relationship between peak current and square root of scan rate (v^1/2^) for MXene and F/M-100. Figure S4. Electrochemical response of MXene, F/M-50, F/M-100, and F/M-150 modified electrodes to 0.5 μM Hg^2+^ in 0.1 M NaAc-HAc buffer solution (pH = 5). Figure S5. Optimization of experimental conditions for Hg^2+^ detection using F/M-100 modified GC electrode: (a) different modification materials, (b) pH value, (c) deposition time, (d) deposition potential. Figure S6. Cyclic voltammetry (CV) response curves of 5 µg/L Hg^2+^ in 0.1 M NaAc-HAc buffer solution (pH = 5.0) at different scan rates (0.05–0.4 V·s^−1^); the inset shows the linear fitting relationship between the oxidation peak potential (E_p_) and the natural logarithm of the scan rate [ln(v)]. (a,c) MXene; (b,d) F/M-100. Figure S7. The square wave anodic stripping voltammetry (SWASV) responses of 0.5 μM Hg^2+^ on the F/M-100 modified glassy carbon electrode in the presence of different interfering substances. Considering the complexity of natural water components, the effects of common anions and cations on the Hg^2+^ signal at the constructed electrochemical sensing interface were investigated. Figure S8. The results of repeated detection of 0.5 μM Hg^2+^ over 14 consecutive days using the F/M-100@PM modified electrode. Figure S9. SWASV response of the F/M-100 modified GCE in a blank groundwater sample. Figure S10. (a) SWASV responses of the F/M-100 modified GCE in groundwater spiked with Hg^2+^ concentrations ranging from 0.2 to 1.0 µM, and (b) the corresponding calibration curve. Table S1. Comparison of the analytical performance of the proposed FePc/MXene (F/M-100) sensor with representative electrochemical sensors for Hg^2+^ detection. Table S2. Results of mercury ion detection and analysis in actual water samples (n = 3). Refs. [14,15,40,41] are cited in the supplementary materials.
