# Supplementary material for: FePc/Mxene-Modified Electrode as a Highly Sensitive Sensing Platform for the Detection of Hg2+ in a Water Environment
Source: Nanomaterials (Basel). 2026 Jun 9;16(12):708. doi: 10.3390/nano16120708 (PMC13305963; doi:10.3390/nano16120708)
Supplement: Supplementary file 1 [file nanomaterials-16-00708-s001.zip › nanomaterials-4332763-supplementary.pdf]

FePc/MXene-Modified Electrode as a Highly Sensitive Sensing Platform for the Detection of Hg^{2+} in a Water Environment

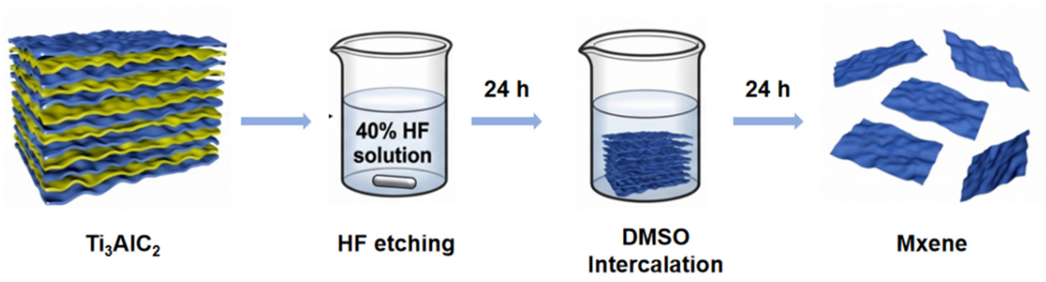

Figure S1. Schematic illustration of the preparation of MXene nanocomposite.

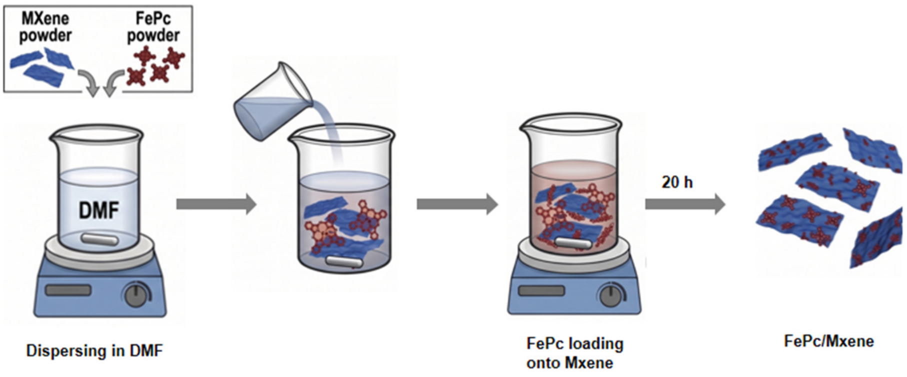

Figure S2. Schematic illustration of the preparation of FePc/MXene-x nanocomposite.

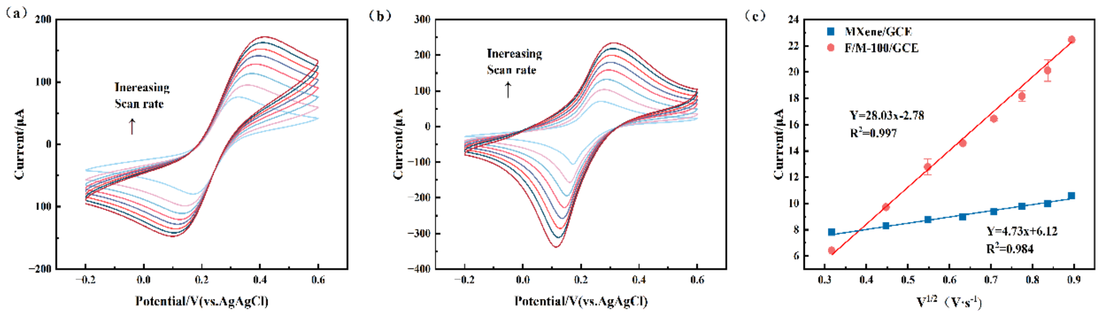

Figure S3. Cyclic voltammetry curves of (a) MXene and (b) F/M-100 in 0.1 M KCl solution containing 5 mM $[Fe(CN)_6]^{3-/4-}$ at different scan rates (0.1–0.8 V/s), and (c) the relationship between peak current and square root of scan rate ($v^{1/2}$) for MXene and F/M-100.

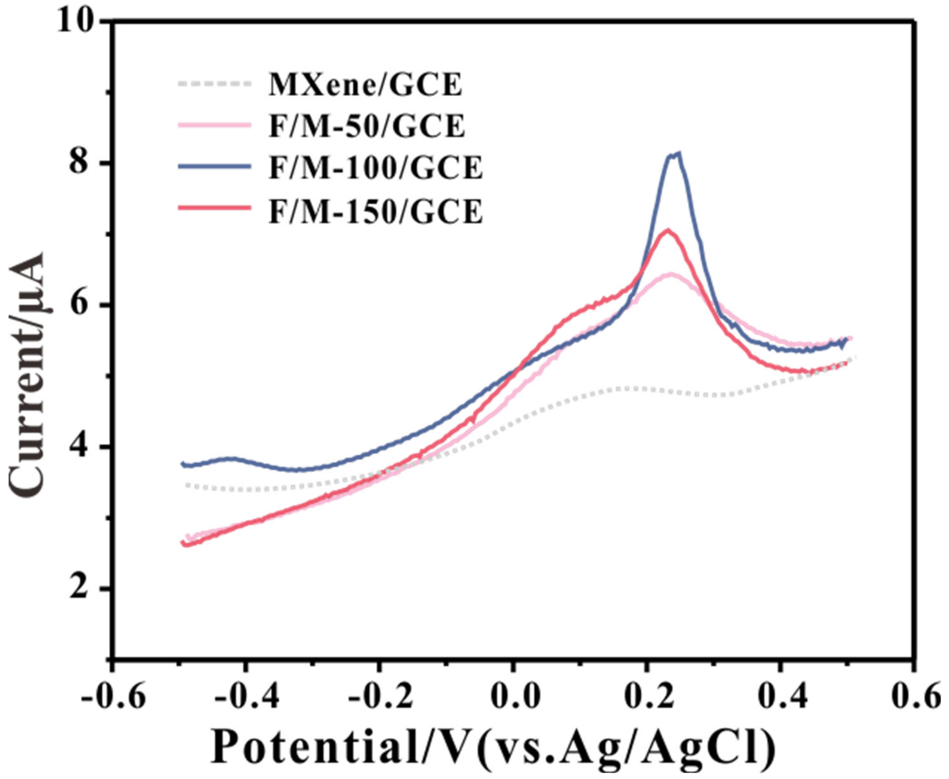

Figure S4. Electrochemical response of MXene, F/M-50, F/M-100, and F/M-150 modified electrodes to 0.5 μM Hg^{2+} in 0.1 M NaAc-HAc buffer solution (pH = 5).

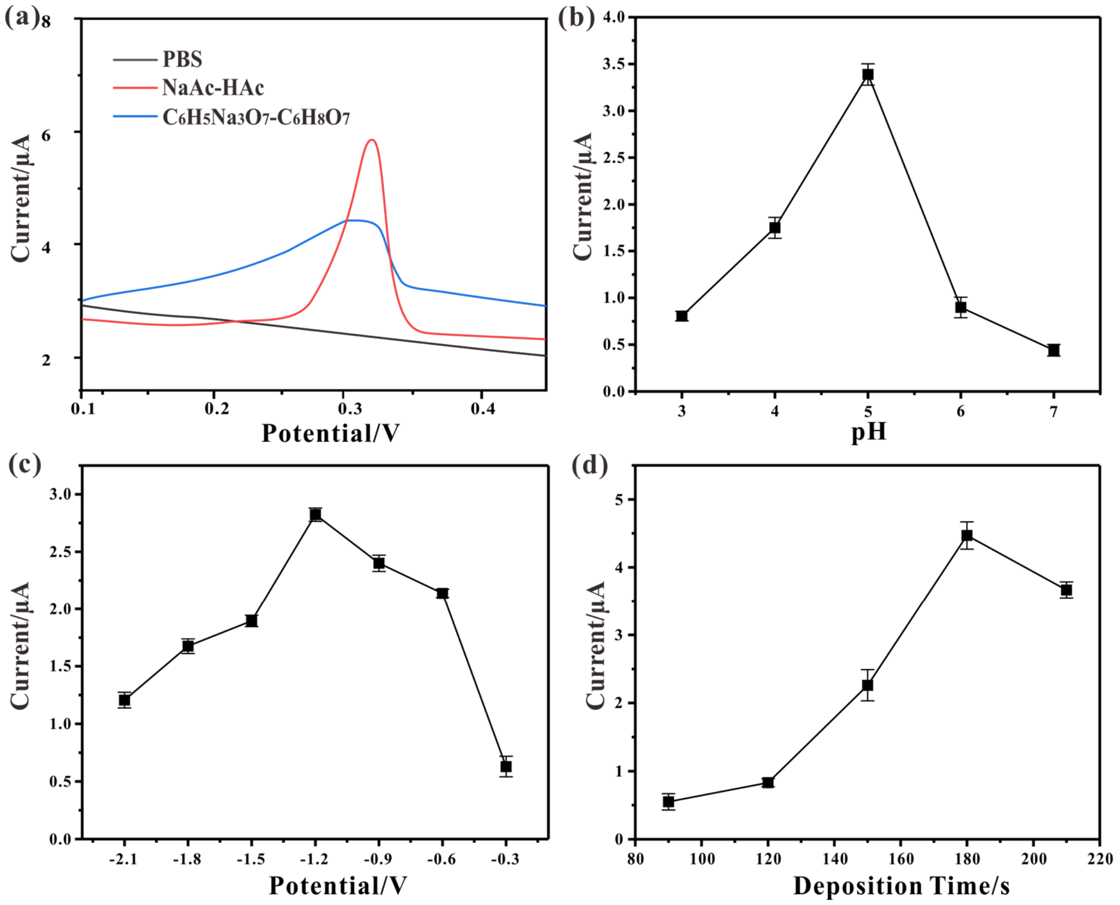

Figure S5. Optimization of experimental conditions for Hg^{2+} detection using F/M-100 modified GC electrode: (a) different modification materials, (b) pH value, (c) deposition time, (d) deposition potential.

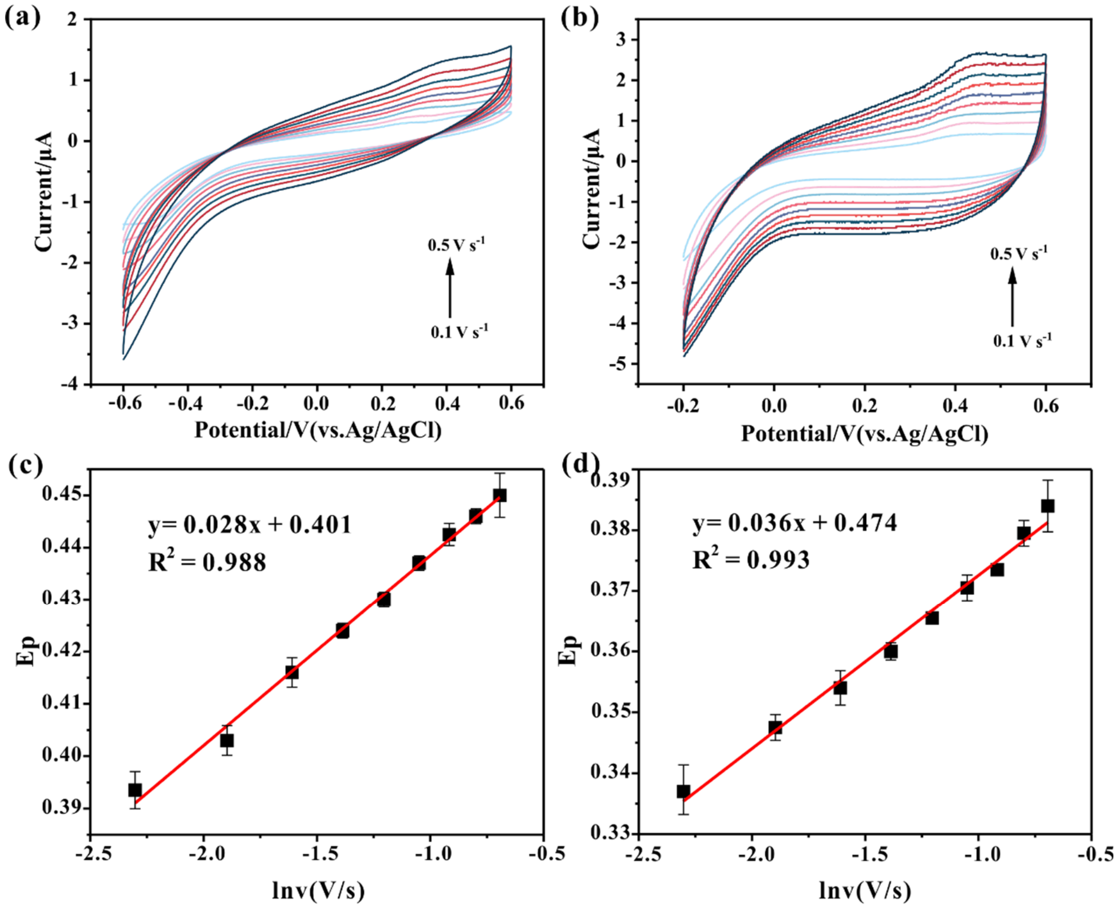

Figure S6. Cyclic voltammetry (CV) response curves of 5 μg/L Hg^{2+} in 0.1 M NaAc-HAc buffer solution (pH = 5.0) at different scan rates (0.05–0.4 $V \cdot s^{-1}$); the inset shows the linear fitting relationship between the oxidation peak potential (E_p) and the natural logarithm of the scan rate [ln(v)]. (a, c) MXene; (b, d) F/M-100.

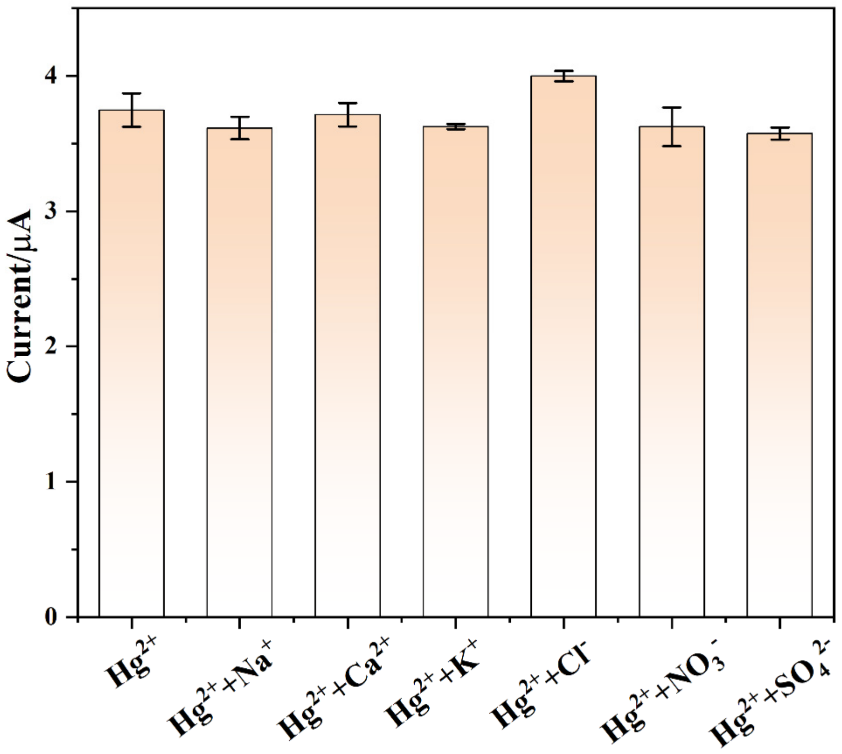

Figure S7. The square wave anodic stripping voltammetry (SWASV) responses of 0.5 μM Hg^{2+} on the F/M-100 modified glassy carbon electrode in the presence of different interfering substances. Considering the complexity of natural water components, the effects of common anions and cations on the Hg^{2+} signal at the constructed electrochemical sensing interface were investigated.

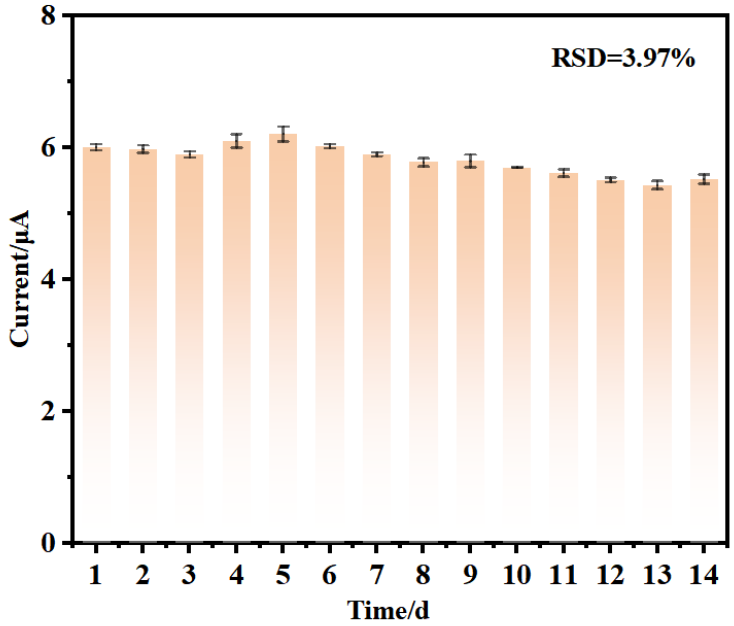

Figure S8. The results of repeated detection of 0.5 μM Hg^{2+} over 14 consecutive days using the F/M-100@PM modified electrode.

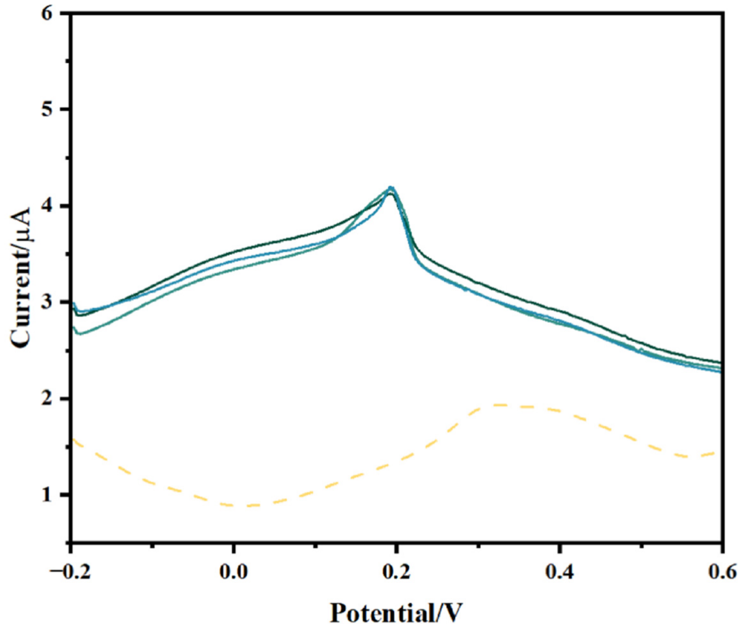

Figure S9. SWASV response of the F/M-100 modified GCE in a blank groundwater sample.

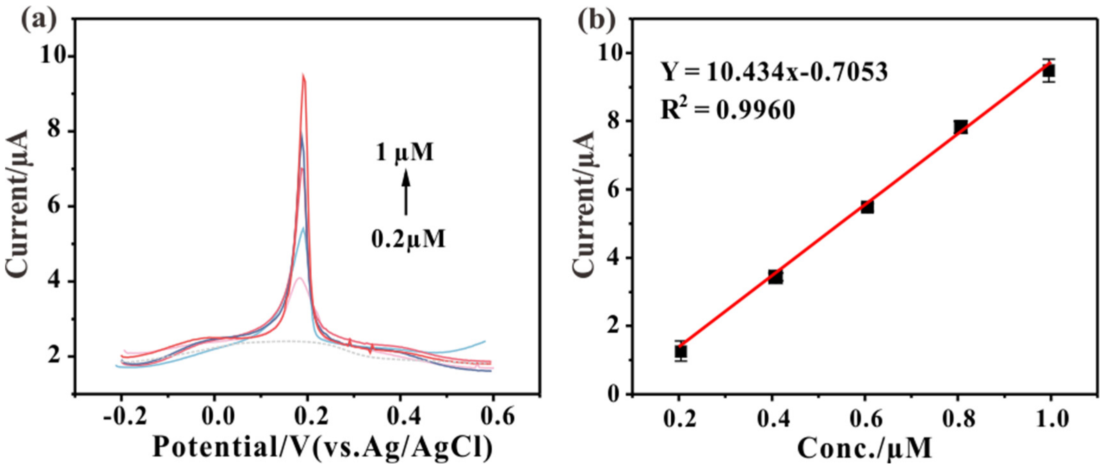

Figure S10. (a) SWASV responses of the F/M-100 modified GCE in groundwater spiked with Hg^{2+} concentrations ranging from 0.2 to 1.0 μM, and (b) the corresponding calibration curve.

Table S1. Comparison of the analytical performance of the proposed FePc/MXene (F/M-100) sensor with representative electrochemical sensors for Hg^{2+} detection.

Electrode Material	Method	Linear Range (μM)	LOD (nM)	Real Sample Type	Recovery (%)	Stability (RSD, %)	Key Advantages / Limitations	References
GCE/SCBB@Ag	DPV	0–0.07 (0–14 ppb)	5.5	-	-	<4.3%	Low cost, easy preparation; relatively narrow linear range	[1]
Cu-MOFs@MnO_2 NSs/SPCE	DPSV	0.5 – 50	30.07	Tap water, Xiangjiang River water	90.20–113.59	1.08%	Good selectivity; moderate LOD	[2]
$BiVO_4$ nanospheres/GCE	SWASV	0-110	1200	-	-	-	Very low LOD; complex synthesis	[3]
AuNPs/graphene-Nafion/GCE	DPV	0.01 – 2.5	0.0036	Laboratory tap water, Drinking water	98.9 – 112.3	0.67% (15 CV cycles)	High conductivity; high cost of gold	[4]
FePc/MXene (F/M-100)/GCE （This work）	SWASV	0.1 – 1.0	5.9	Lake water (HuBing Pond), Groundwater	-	2.03% (n=6) 2.49% (7days)	High sensitivity, excellent stability; moderate linear range	This work

Table S2. Results of mercury ion detection and analysis in actual water samples (n = 3).

Sample	Added(μM)	Measured(μM)	Recovery	Relative standard deviation(%)
Hubing Tang	0.2	0.21	105.00%	2.31
	0.4	0.39	97.50%	2.21
	0.6	0.63	105.00%	3.53
	0.8	0.79	98.75%	2.86
	1	1.03	103.00%	1.86
Groundwater	0.2	0.22	95.00%	2.34
	0.4	0.38	95.00%	3.01
	0.6	0.62	103.33%	2.75
	0.8	0.79	98.75%	2.75
	1	1.01	101.00%	1.75

References:

[1]Lo M, Tang M, Faye D, et al. Silver-modified sugarcane bagasse biochar-based electrode materials for the electrochemical detection of mercury ions in aqueous media[J]. Electrochimica Acta, 2025: 147214.

[2]Liu J, Liu S, Shi J, et al. A Cu-MOFs@ MnO_2 nanocomposite-based ratiometric electrochemical sensor for simultaneous detection of Pb^{2+} and Hg^{2+}[J]. Microchemical Journal, 2026: 116742.

[3]Madhivanan K, Atchudan R, Arya S, et al. Simultaneous electrochemical detection of heavy metal ions using a sol–gel synthesized $BiVO_4$ nanosphere modified electrode and its antimicrobial activity[J]. Nanoscale Advances, 2025, 7(11): 3432-3448.

[4]Li Z, Miao X, Xing K, et al. Ultrasensitive electrochemical sensor for Hg^{2+} by using hybridization chain reaction coupled with Ag@ Au core–shell nanoparticles[J]. Biosensors and Bioelectronics, 2016, 80: 339-343.
